# Adipogenic differentiation was inhibited by downregulation of PPARγ signaling pathway in aging tendon stem/progenitor cells

**DOI:** 10.1186/s13018-021-02720-y

**Published:** 2021-10-18

**Authors:** Fan Lai, Jingjing Wang, Hong Tang, Xuting Bian, Kang Lu, Gang He, Pan Huang, Juan Liu, Mei Zhou, Jian Liu, Xu Tao, Kang-lai Tang

**Affiliations:** 1 Department of Sports Medicine Center, Southwest Hospital, Army Medical University, Chongqing, 400038 China; 2 Department of Blood Transfusion, Xinqiao Hospital, Army Medical University, Chongqing, 400037 China; 3 Health Team of Troop No. 96785 of PLA, Luoyang, 471300 China

**Keywords:** Tendon stem/progenitor cells, Aging, Adipogenesis, PPAR gamma signaling

## Abstract

**Background:**

Tendon stem/progenitor cells (TSPCs) play a vital role in tendon repair and regeneration. Previously we found more adipocytes accumulated in the patellar tendon injury sites in aging rats compared with the young ones, of which the mechanism is still unknown. Here, we want to identify whether erroneous differentiation of TSPCs by aging accounts for the adipocyte accumulation.

**Methods:**

TSPCs from young and aging rats were isolated and propagated. Both young and aging TSPCs were induced to differentiate into adipocytes, and Oil red O staining, quantitative real-time polymerase chain reaction (qRT-PCR), western-blot and immunofluorescent staining were used to evaluate the capability of TSPCs. RNA sequencing was utilized to screen out different genes and signaling pathways related to adipogenesis between young and aging TSPCs.

**Results:**

The Oil red O staining showed there were more adipocytes formed in young TSPCs. Besides, adipogenic markers perilipin, peroxisome proliferator-activated receptor γ (PPARγ), CCAAT/enhancer-binding proteins alpha (C/EBPα) and Fatty acid-binding protein 4 (FABP4) were elevated both at gene and protein level. PPARγ signaling pathway was selected as our target via RNA sequencing. After adding the signaling activators, Rosiglitazone maleate (RM), inhibited adipogenesis of aging TSCs was reversed.

**Conclusions:**

In conclusion, aging inhibited adipogenesis of TSPCs by down‐regulating PPARγ signaling. It is not likely that the adipocyte accumulation in aging tendon during repair was due to the aging of TSPCs. This may provide new targets for curing aging tendon injuries or tendinopathies.

## Background

Tendon injuries are some of the most common orthopedic problems that cause severe pain, disability and financial burden [1]. Studies have shown that with the aging of individuals, the structure and function of tendons have undergone significant changes, which are closely related to the functional changes of Tendon stem/progenitor cells (TSPCs) in the tendon [2]. TSPCs were first isolated and extracted by Bi in 2007[3]. They are derived from tendon tissue and showed standard mesenchymal stem cell (MSC) characteristics, with expression of typical MSC surface antigens, self-renewal, colony-forming, and tri-lineage differentiation potential [4–6]. At present, many studies have proved that TSPCs participate in tendon repair and regeneration and play a key role [7–9]. In our previous study, we found that there were more adipocytes gathered in the process of tendon repair in aging rats than in young rats, which impaired the effect of tendon regeneration and repair, but the specific mechanism has not been elucidated. One possible hypothesis is that in the process of tendon repair, TSPCs involved in adipogenic differentiation should have differentiated into tenocytes to promote tendon repair. As a result, tendon regeneration was inevitably impaired.

Studies have found that TSPCs express more tenogenic markers than other sources of mesenchymal stem cells, and they have a tendency to spontaneously differentiate into tendon cells in vivo, which is beneficial to the repair of tendon or tendon enthesis [10, 11]. However, more and more evidences show that TSPCs also play different roles under certain conditions. For example, RUI et al. found that the heterogeneous differentiation of TSPCs after changes in mechanical and biological environment led to calcified tendon disease [12]; another study found that the presence of tenomodulin can prevent TSPCs from differentiating into adipocytes, thus inhibiting the accumulation of adipocytes in the early stage of tendon repair [13].

Senescence begins with the degeneration of cells, which is a process in which the physiological functions of tissues, organs and the whole body degenerate gradually with time. Han et al. found that p16 inhibits tendon differentiation of TSPCs through microRNA-217 and its target gene EGR1 in aged rat TSPCs [14]. Another study found that the stem cell plasticity and adipogenic differentiation ability of adipose-derived stem cells isolated from older female rabbits were significantly lower than those of young rabbits [15]. In this regard, whether the aging of TSPCs leads to the change of their adipogenic ability, and whether it indirectly leads to the accumulation of adipocytes in the repaired tendons of aged rats. The answers to these questions may provide us with new therapeutic targets for the treatment of age-related tendon injuries and tendon diseases.

In this study, we conducted oil red O staining, gene and protein detection after induction of adipogenic differentiation on TSPCs in vitro. It was demonstrated that the potential of adipogenic differentiation of TSPCs isolated from aging rat tendons was significantly inhibited compared with those isolated from young rat tendons. Through gene sequencing, it was found that the decrease of adipogenic differentiation potential of TSPCs was due to the inhibition of PPARγ signaling pathway in aging rats. These results suggest that the aging of TSPCs might not be the reason for the accumulation of adipocytes during patellar tendon repair in aging rats.

## Methods

### Cell isolation and culture

The previous method was used to isolate TSPCs [11]. The patellar tendons of male Sprague Dawley rats at 3 months (young group) and 24 months (aging group) were taken. All rats were provided by the laboratory animal center of Army Medical University. Three rats in each group were killed by CO_2_ asphyxia (flow rate of CO_2_: 30% of chamber volume per minute), and the patellar tendon was dissected, only the intermediate tissue was collected, and the surrounding connective tissue was carefully removed. The collected tissue was washed with 0.01 mol/L phosphate buffered saline (PBS) (Solarbio, China) and then cut as small as possible. Then, at 37 °C the tissue was digested in a 3 mg/mL type I collagenase (sigma Aldrich) culture flask for 3 h. After that, the cells were filtered by a 70-μm nylon mesh to produce a single-cell suspension. The released cells were washed with 0.01 mol/ L PBS and centrifuged for 5 min in 50 g, then resuspended in fresh medium containing Dulbecco’s modified Eagle’s medium (DMEM) (GIBCO) and 15% fetal bovine serum (FBS) (Invitrogen, Carlsbad, CA) and 1% penicillin/streptomycin (Invitrogen, Carlsbad, CA). TSPCs were cultured in a cell incubator at 37 °C and 5% CO_2_ and passaged when the cells reached about 70% confluence with the medium changed every 3 days. The third to fifth generation cells were used in all the experiments. Young and aging TSPCs were inoculated on 24 well plates with cell slides for cell immunofluorescence staining and 6 well plates for protein and RNA extraction and oil red O staining, respectively. All experiments were repeated with at least three independent biological replicates.

### β-galactosidase staining

The β-galactosidase (β-gal) assay was performed using the Senescence-β-Galactosidase Kit (Beyotime). Young and aging TSPCs were plated on 6-well plates and incubated for 48 h. Cells were incubated with the kit’s staining mixture for 16 h at 37 °C without CO_2_. The percentages of β-gal-positive cells were calculated by counting positive cells and all the cells in nine random microscopic fields. Images were captured by an Olympus CKX53 inverted phase-contrast microscope (× 4 objectives).

### Adipogenic induction

The young and aging TSPCs were seeded on a 6-well cell culture plate with a density of 50,000/well, cultured with DMEM containing 10% FBS (Invitrogen, Carlsbad, CA), and the solution was changed every three days. When the cells confluenced to 100%, we began to carry out adipogenic induction culture on the two groups of TSPCs with the adipogenesis induction medium (Cyagen, RASMX-90031, China). First, we sucked out the complete medium, replaced with the adipogenic induction medium solution A (SD Rat Bone Marrow Mesenchymal Stem Cell Adipogenic Differentiation Basal Medium A 175 mL, SD Rat Bone Marrow Mesenchymal Stem Cell Adipogenic Differentiation Fetal Bovine Serum 20 mL, Penicillin–Streptomycin 2 mL, Glutamine 2 mL, Insulin 400 μL, IBMX 200 μL, Rosiglitazone 200 μL, Dexamethasone 200 μL), and after three days of induction, we changed it into adipogenic induction medium solution B (SD Rat Bone Marrow Mesenchymal Stem Cell Adipogenic Differentiation Basal Medium B 175 mL, SD Rat Bone Marrow Mesenchymal Stem Cell Adipogenic Differentiation Fetal Bovine Serum 20 mL, Penicillin–Streptomycin 2 mL, Glutamine 2 mL, Insulin 400 μL) and it was cultured for 24 h, then solution B was sucked out, and then replaced with adipogenic induction medium solution A to continue to induce. After three times of alternation between solution A and solution B, solution B was used to maintain the culture for 1 week until the lipid droplets became large and round enough. Solution B was changed every three days during the culture. The TSPCs in each group were used to conduct qRT-PCR, western-blot and oil red O staining, respectively.

### Oil red O staining

After the adipogenic differentiation induction, the medium for adipogenic differentiation induction in the six-well plate was sucked out and rinsed with 1 × PBS twice. 2 mL 4% neutral formaldehyde solution was added to each well and fixed for 30 min. The neutral formaldehyde solution was sucked and rinsed twice with 1 × PBS. An oil red O stain kit (Solarbio, G1262) was used to conduct this experiment. 1 mL oil red O staining working solution was added to each well for 30 min (preparation method of working solution: oil red O storage solution: distilled water = 3:2, mix well and filter with neutral filter paper). The oil red O dye was sucked and rinsed with 1 × PBS for 3 times. Then the hematoxylin was used to dye the nucleus. The effect of lipid staining was observed under a microscope. Four photographs were randomly taken in each well (3 wells in each group) and semi-quantitative analysis was carried out by software imageJ (National Institutes of Health).

### Quantitative reverse transcription polymerase chain reaction

The gene expression levels of adipogenic markers and the molecules on the PPARγ signal pathway were performed by quantitative reverse‐transcription PCR (qRT‐PCR). Total RNA was extracted by TRIzol reagent (Takara, Dalian, China) from cells according to the protocol. The superscript III first‐strand synthesis kit (TaKaRa) was used to synthesize complementary DNA (cDNA) from total RNA. The SYBR Green RT‐PCR kit (Takara) and ABI Prism 7900 Sequence Detection System (PE Applied Biosystems, Foster City, CA) were utilized for qPCR. Total cDNA of each sample (5 μl) was amplified in a final volume of 25 μL of reaction mixture. Housekeeping gene GAPDH was used as the internal control. PCR primer sequences are shown in Table 1.

**Table 1:** Primers sequence for PCR analysis

Primer	Sequence
Perilipin	Forward 5′-GCCTCTGTGTGCAATGCCTA-3′
	Reverse, 5′-GAGCCGGGATCTTTTCCTCC-3′
Slc27a6	Forward 5′-CCTATGAGGATGTGGACAAGAGG-3′
	Reverse, 5′-CCTGATATGTTTCGGGAGGCT-3′
RXRA	Forward 5′-GCACCCTGAGTTCTCCCATC-3′
	Reverse, 5′-ATAGCGCAGATGTGCTTGGT-3′
CD36	Forward 5′-AGATGCAGCCTCCTTTCCAC-3′
	Reverse, 5′-GCGTTGGCTGGAAGAACAAA-3′
FABP3	Forward 5′-TCAAGTCGGTCGTGACACTG-3′
	Reverse, 5′-GCCTCCTTCTCGTAAGTCCG-3′
OLR1	Forward 5′-CATGGGCCCTTTAACTGGGA-3′
	Reverse, 5′-GAAACGCCCCTGGTCCTAAA-3′
PPARγ	Forward 5′-CCTTTACCACGGTTGATTTCTC-3′
	Reverse, 5′-GGCTCTACTTTGATCGCACTTT-3′
C/EBPα	Forward 5′-TACCTGGGCTACCAGGCGA-3′
	Reverse, 5′-CGCGCCGCATCTTGTACTC-3′
FABP4	Forward 5′-CGAGATTTCCTTCAAACTGGG-3′
	Reverse, 5′-TCTTGTAGAAGTCACGCCTTTC-3′
GAPDH	Forward 5′-TGACTTCAACAGCAACTC-3′
	Reverse, 5′-TGTAGCCATATTCATTGTCA-3′

### Western blot

In the part of senescence assays, the third generation of young and aging TSPCs were collected to examine the expressions of senescence markers β-galactosidase and p16^ink4a^. In the part of adipogenic-related markers examination, we selected the cells cultured for 14 days with induction medium to detect the expression level of adipogenic markers and related protein molecules in PPARγ signaling pathway. After sucking out the culture medium from the culture dish, we washed the cells with PBS twice and added the protein lysate to extract the total protein, then measured the concentration of the protein sample by BCA (Bestbio). The protein samples (20 μg/lane) were separated on a 12% SDS–polyacrylamide gel at 120 V for 50 min, and then transferred to the nitrocellulose membrane at 120 V for 70 min. Tris-buffer saline containing 0.1%Tween-20 and 5% fat-free milk was used to block the membrane for 2 h at room temperature. Then the membranes were incubated with the first antibodies (anti-β-galactosidase: Bio-Vision (1:500), anti-p16^ink4a^: Bioss (1:500), anti-Perilipin: abcam (1:500), anti-PPARγ abcam (1: 500), anti-C/EBPα: Santa Cruz Biotechnology (1: 500), anti-FABP4: abcam (1:1000), anti-Angptl-4: bioss (1:500), anti-Slc27a6: bioss (1:500), Shake at 4 °C overnight. Before adding the second antibody (a peroxidase‐conjugated goat anti‐rabbit immunoglobulin G (1:1000; Santa Cruz Biotechnology) and chicken anti‐mouse immunoglobulin G (1:1000; Santa Cruz Biotechnology)) for 2 h. The enhanced chemiluminescence detection kit (GE Healthcare, Wuxi, China) was used to visualize and capture the protein images.

### Immunofluorescence

Cells placed on slides were fixed using 4% paraformaldehyde for 30 min. After washing twice with PBS, the cells were punched and blocked with 0.1% Triton 100 and 5% bovine serum albumin for 1 h. After washing with PBS, the cells were incubated with primary antibodies (anti-Perilipin abcam (1:500), anti-FABP4 abcam (1:1000), and anti-C/EBPα Santa Cruz Biotechnology (1: 500)) at room temperature for 3 h. After washing with PBS, the cells were incubated with secondary antibodies (Santa Cruz Biotechnology) for 30 min. After staining with DAPI (Sigma-Aldrich) for 5 min, the cells were observed and photographed under a laser scanning confocal microscope (Olympus IX70, Tokyo, Japan).

### mRNA‐seq analysis

The mRNA-Seq experiments were performed by Novogene (Beijing, China). mRNA-seq library is prepared for sequencing using standard Illumina protocols as it was previously described [16]. Briefly, total RNAs from rat young and aging TSPCs with adipogenic induction are isolated using TRIzol reagent (Invitrogen) and then the total RNAs were treated with RNase-free DNase I (New England Biolabs, MA, USA), to avoid any genomic DNA contamination. mRNA extraction is performed using Dyna-beads oligo (dT) (Invitrogen Dynal). Superscript II reverse transcriptase (Invitrogen) and random hexamer primers are used to synthesize the double-stranded complementary DNAs. The cDNAs are then fragmented by nebulization. The mRNA-seq library was created by following the standard Illumina protocol using NEBNext® UltraTM RNA Library Prep Kit for Illumina® (NEB, USA). RNA integrity was assessed using the RNA Nano 6000 Assay Kit of the Bioanalyzer 2100 system (Agilent Technologies, CA, USA). The sequencing kit TruSeq PE Cluster Kit v3-cBot-HS (Illumia, NEB, USA) was used for sequencing. Briefly, mRNA was purified from total RNA using poly-T oligo-attached magnetic beads. Fragmentation was carried out using divalent cations under elevated temperature in NEBNext First Strand Synthesis Reaction Buffer (5X). First strand cDNA was synthesized using random hexamer primer and M-MuLV Reverse Transcriptase (RNase H-). Second strand cDNA synthesis was subsequently performed using DNA Polymerase I and RNase H. Remaining overhangs were converted into blunt ends via exonuclease/polymerase activities. After adenylation of 3′ ends of DNA fragments, NEBNext Adaptor with hairpin loop structure were ligated to prepare for hybridization. In order to select cDNA fragments of preferentially 250–300 bp in length, the library fragments were purified with AMPure XP system (Beckman Coulter, Beverly, USA). Then 3 µl USER Enzyme (NEB, USA) was used with size-selected, adaptor-ligated cDNA at 37 °C for 15 min followed by 5 min at 95 °C before PCR. Then PCR was performed with Phusion High-Fidelity DNA polymerase, Universal PCR primers and Index (X) Primer. At last, PCR products were purified (AMPure XP system) and library quality was assessed (the effective concentration of library is higher than that of 2 nM) on the Agilent Bioanalyzer 2100 system.

### Bioinformatics

Raw data (raw reads) of fastq format were firstly processed through in-house perl scripts. Index of the reference genome was built using Hisat2 v2.0.5 and paired-end clean reads were aligned to the reference genome using Hisat2 v2.0.5. We selected Hisat2 as the mapping tool for that Hisat2 can generate a database of splice junctions based on the gene model annotation file and thus a better mapping result than other non-splice mapping tools. The mapped reads of each sample were assembled by StringTie (v1.3.3b). FeatureCounts v1.5.0-p3 was used to count the reads numbers mapped to each gene. Differential expression analysis of two conditions/groups (two biological replicates per condition) was performed using the DESeq2 R package (1.16.1). The resulting P-values were adjusted using the Benjamini and Hochberg’s approach for controlling the false discovery rate. Genes with an adjusted P-value < 0.05 found by DESeq2 were assigned as differentially expressed. Gene Ontology (GO) enrichment analysis of differentially expressed genes was implemented by the clusterProfiler R package, in which gene length bias was corrected. GO terms with corrected Pvalue less than 0.05 were considered significantly enriched by differential expressed genes. KEGG is a database resource for understanding high-level functions and utilities of the biological system, such as the cell, the organism and the ecosystem, from molecular-level information, especially large-scale molecular datasets generated by genome sequencing and other high-through put experimental technologies (http://www.genome.jp/kegg/). We used clusterProfiler R package to test the statistical enrichment of differential expression genes in KEGG pathways.

### Statistical analysis

All values are expressed as the mean ± standard deviation (SD). Student’s t-test was used to compare means between two groups. Multiple comparisons were made using one-way analysis of variance followed by Fisher’s test. A *p*-value < 0.05 was considered statistically significant.

## Results

### Higher senescence markers β-galactosidase and p16^ink4a^ were expressed in aging TSPCs

**Fig. 1:**
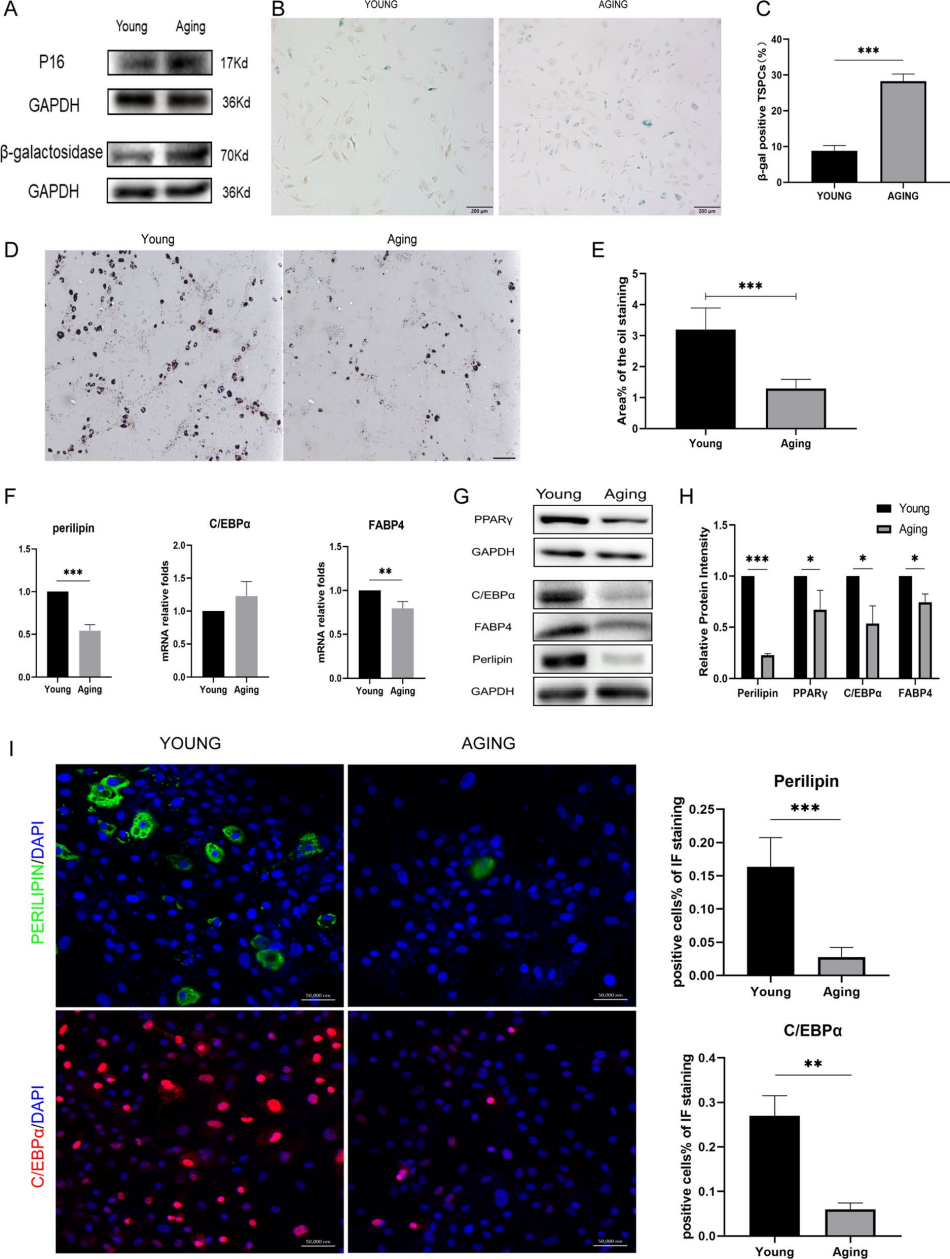
Adipogenesis was inhibited in aging TSPCs. **A** western-blot results of senescence markers β-galactosidase and p16^ink4a^ in young and aging TSPCs. **B** β-gal staining of young and aging TSPCs. **C** quantitative analysis of the β-gal positive staining TSPCs (%). **D** Oil red O staining of young and aging TSPCs after 3 weeks of adipogenic induction showed much less lipid droplets in aging group. Black bars: 50 μm. **E** Quantitative analysis of the lipid droplets showed significant difference between two groups. **F** qPCR analysis of adipogenic markers (perilipin, C/EBPα and FABP4) expression of young and aging TSPCs after 1 week of adipogenic induction showed significant difference in the expression of perilipin and FABP4. **G**, **H** western blot analysis of adipogenic markers (PPARγ, perilipin, C/EBPα and FABP4) expression of young and aging TSPCs. Expression of PPARγ, C/EBPα, FABP4 and perilipin were downregulated in aging TSPCs. **I** Immunofluorescence staining of adipogenic markers (Perilipin and C/EBPα) expression of young and aging TSPCs. There were more positive cells in young group both on the expression of Perilipin and C/EBPα. *p < .05; **p < .01; ***p < .001; ****p < .000. These experiments were repeated for 3 times

It was reported in previous researches that aging TSPCs have a significantly higher p16 ^ink4a^ and β-galactosidase expression level [2, 14]. To confirm that we detected the expressions of p16 ^ink4a^ and β-galactosidase by western-blot and stained the β-galactosidase in young and aging TSPCs. As is shown in Fig. 1A, there were higher expressions of p16 ^ink4a^ and β-galactosidase in aging TSPCs. As is shown in Fig. 1B, C, the proportion of positive β-galactosidase cells in aging TSPCs was significantly higher than that in young ones.

### The adipogenic differentiation was inhibited in aging TSPCs

In order to explore whether the accumulation of adipocytes in aging tendon was caused by the change of adipogenic ability of TSPCs during aging, we conducted adipogenic induction culture of TSPCs in vitro. First of all, through oil red O staining, we intuitively found that the number of lipid droplets in aging TSPCs after adipogenic induction was significantly lower than that in young TSPCs (Fig. 1D), and the quantitative analysis (Fig. 1E) also showed that there was a significant difference in the number of lipid droplets in young and aging TSPCs. In qRT-PCR results, we found that Perilipin and FABP4 were significantly reduced in aging rat TSPCs after adipogenesis induction (Fig. 1F), while the corresponding content of Perilipin, C/EBPα and FABP4 in aging TSPCs were significantly reduced at the protein level (Fig. 1G, H). The similar results were shown by the Immunofluorescence staining (Fig. 1I).

### RNA‐seq analysis of gene expression profile of young and aging TSCs

**Fig. 2:**
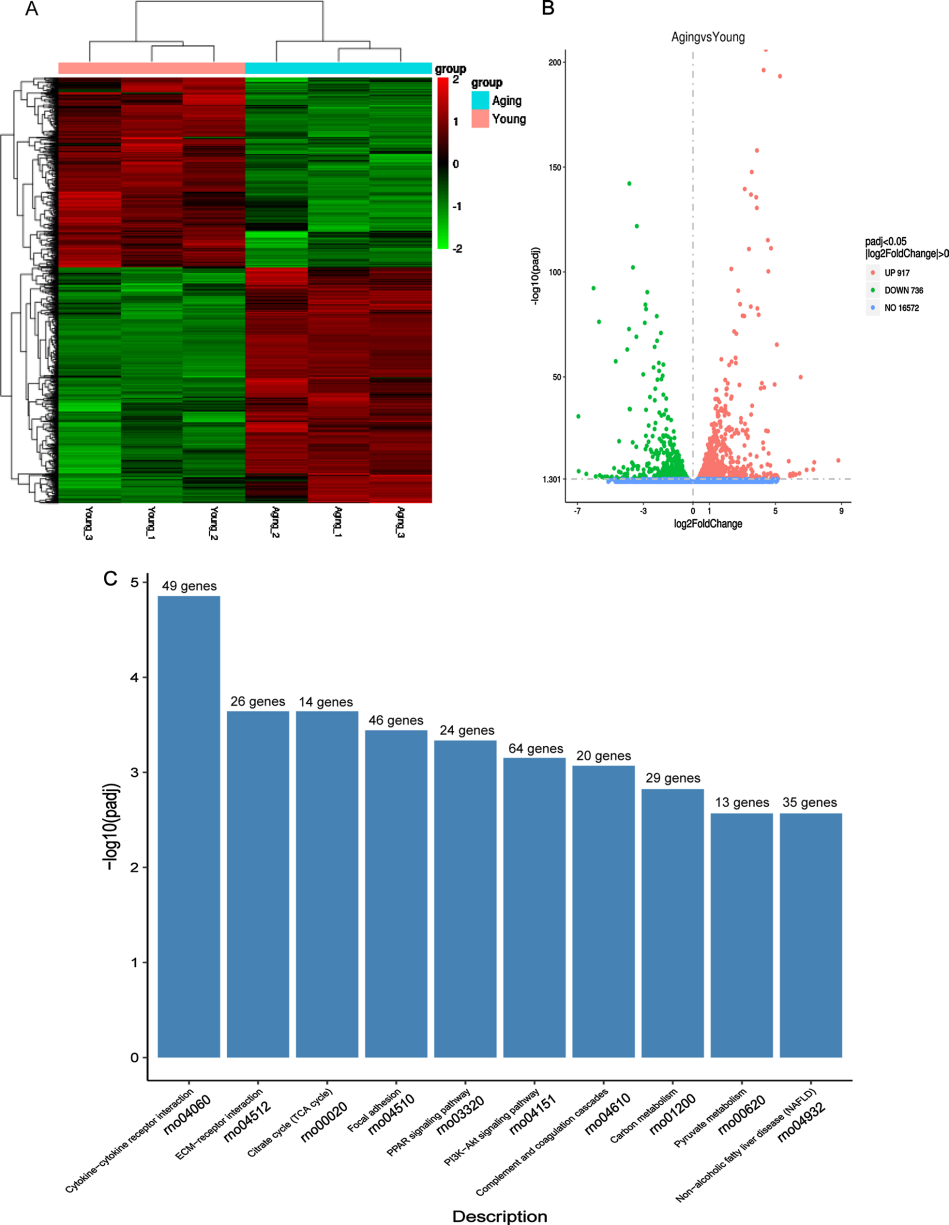
RNA-seq analysis of gene expression profile of young and aging TSPCs with adipogenic induction. **A** Heatmap depicting expression levels of genes between young and aging TSPCs with adipogenic induction. In total, 1653 genes (917 genes were up-regulated and 736 genes were down-regulated) of TSCs were differentially expressed between young and aging TSPCs. **B** Volcano map of the differentially expressed genes of TSPCs between young and aging group. **C** Top 10 enriched signaling pathways analyzed by the KEGG analysis

In order to analyze the potential causes of decreased adipogenic differentiation ability caused by aging in vitro, RNA sequencing was used to observe the changes of TSPCs expression profile. According to the results of heat map and volcanic map (Fig. 2A, B), there were 1653 genes with more than twice difference between young and aging TSPCs, among which 917 genes were up-regulated and 736 genes were down-regulated. Among the 10 pathways with the greatest differences between the younger and aging groups, KEGG results showed that lipid-related pathways included the ‘PPARγ signaling pathway’ (rno03320)and the ‘PI3K-Akt pathway’ (rno04151) (Fig. 2C).

### PPARγ signaling pathway was down-regulated in aging TPSCs

**Fig. 3:**
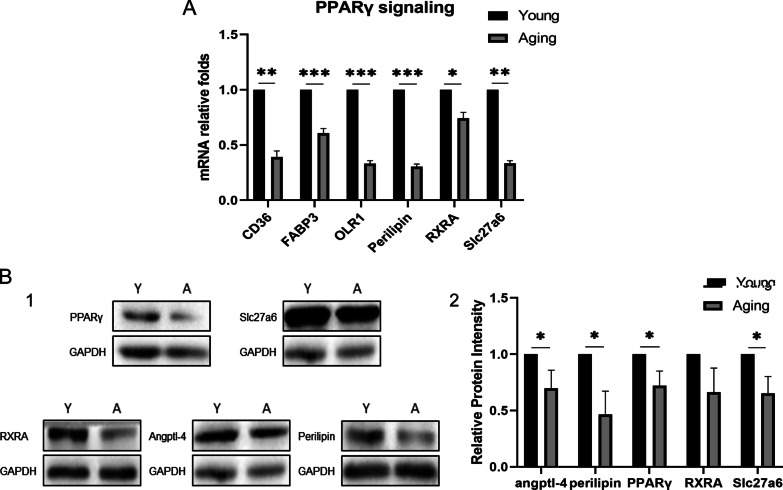
PPARγ signaling pathway was down-regulated in aging TPSCs. **A** qPCR analysis of the PPARγ signaling pathway molecules showed that CD36, FABP3, OLR1, perilipin, RXRA, Slc27a6 were decreased in aging TSPCs. **B** The expression of PPARγ, Slc27a6, RXRA, Angptl-4 and perilipin at protein level were downregulated. *p < .05; **p < .01; ***p < .001; ****p < .000. The qPCR experiment was repeated for 5 times and the western blot experiment were repeated for 5 times

In order to determine the exact pathway affecting the lipid formation of TSPCs in aging rats, we verified the expression of the pathway molecules according to the sequencing results, and found that the gene expression of molecules in the PPARγ signaling pathway, such as CD36, FABP3, OLR1, Perilipin, RXRA and Slc27a6 were significantly decreased (Fig. 3A). The results of western blot showed that the expression of PPARγ, Slc27a6, RXRA, angptl-4 and perilipin were lower in young (Y) TSPCs compared with aging (A) TSPCs (Fig. 3B). The qRT-PCR and western blot results demonstrated that the PPARγ signaling pathway was inhibited in the aging rat TSPCs.

### The adipogenesis of aging TSCs was inhibited through downregulating PPARγ signaling pathway

**Fig. 4:**
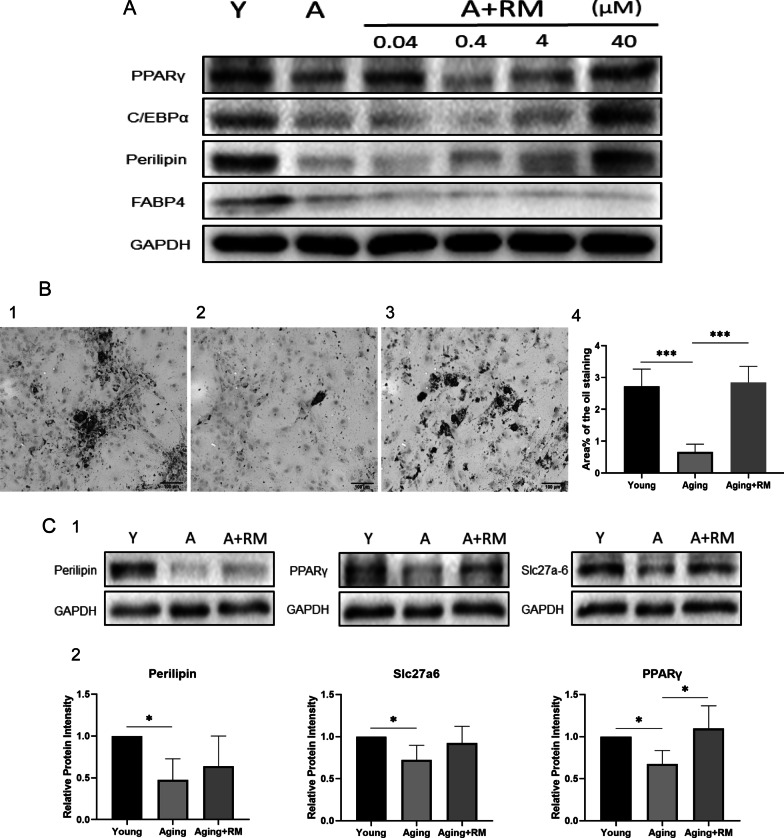
The adipogenesis of aging TSCs was inhibited through downregulating PPARγ signaling pathway. **A** western blot analysis of the adipogenic markers after adding 4 different concentration of Rosiglitazone maleate (RM) in aging TSPCs for 2 weeks. **B** Oil red O staining of young (**B1**), aging (**B2**) and aging with RM (**B3**) TSPCs, (**B4**) The lipid droplets increased in aging TSPCs with RM. **C** The expression of Perilipin, PPARγ and Slc27a6 in young, aging and aging with RM TSPCs. (**B1**) The decreased expression of Perilipin, PPARγ and Slc27a6 were reversed after adding the specific agonist of PPARγ pathway RM. (**B2**) Quantitative analysis of the Western blot results. Black bar: 100 μm. *p < .05; **p < .01; ***p < .001. These experiments were repeated for 5 times

To verify whether PPARγ pathway is the key influencing factors for aging TSPCs differentiating into adipocytes, we added the specificity agonist of PPARγ pathway Rosiglitazone maleate (RM) with 4 different concentrations (0.04, 0.4, 4, 40 μM), in the induction medium. Firstly, western blot was conducted to detect the expression of adipogenic related markers (PPARγ, C/EBPα, perilipin, FABP4). We found that the expression of PPARγ, C/EBPα, perilipin were elevated in a dose-dependent manner, and there was the highest expression with the concentration of 100 ng/mL of Rosiglitazone maleate (Fig. 4A). Then Oil red O staining and the qRT-PCR were conducted, it was revealed that after adding Rosiglitazone maleate agonist with the concentration of 100 ng/mL into the induction medium of aging TSPCs, there had been a marked increase in the number of lipid droplets (Fig. 4B-1–4). At the same time, The expression of PPARγ, perilipin and Slc27a6 (Fig. 4C-1) in the PPARγ signaling pathway in aging TSPCs were up-regulated compared with the aging TSPCs without Rosiglitazone maleate, though only the expression of PPARγ was significantly different (Fig. 4C-2).

## Discussion

Fatty infiltration and ectopic ossification are common phenomena of tendon tissue after injury [12, 13, 17–19]. Inhibiting the accumulation of adipocytes during tendon repair is one of the key problems to promote tendon repair. TSPCs are a kind of mesenchymal stem cells derived from tendon tissue which have the potential of self-renewal and multi-directional differentiation [3, 4], but because they come from tendon tissue, they express more markers related to tendon differentiation and have a strong trend of tendon differentiation [10, 11]. TSPCs residing in tendons are responsible for tissue homeostasis and for the maintenance and repair of the tissue after trauma [9]. However, there is no unified understanding of the effect of aging on the differentiation of mesenchymal stem cells. Though some studies have found that young and aging MSCs have similar differentiation potential [20]. It has also been reported that with aging, the differentiation ability of stem cells will gradually decrease [21, 22]. And what is more interesting is that Zhou etc. found that the tenogenic differentiation decreased and adipocyte differentiation including adipogenic markers increased in aged rat TSPCs [23] in an in vitro study. We believe that it is of great significance to reveal the exact effect of aging on differentiation of TSPCs, which may help us to understand the potential mechanism that how TSPCs participate in tendon healing and to solve the clinical problem of tendon repair in the elderly. In this regard, the purpose of this study is to clarify the effect of aging on adipogenic differentiation of TSPCs and its exact mechanism, and to determine whether the aging of TSPCs leads to the accumulation of adipocytes during the repair process of tendon injury in the elderly.

In this study, TSPCs were isolated from patellar tendons of male Sprague Dawley rats of 3 months (young group) and 24 months (aging group). In order to verify the changes of adipogenic differentiation ability in aging TSPCs, we conducted oil red O staining on young and aging TSPCs after adipogenic induction in vitro. We found that the red lipid droplets were formed in both groups, but the number of lipid droplets and perilipin, which indirectly reflected the number of lipid droplets, were significantly decreased in the aging TSPCs. At the same time, the adipogenic markers Perilipin, FABP4, C/EBPα were significantly decreased. All the results revealed that aging inhibited the adipogenesis of TSPCs.

It has been reported that signal transduction pathways, including AMPK, JAK-STAT and PPARγ, can affect adipogenic differentiation of adipose precursors or mesenchymal stem cells. Yao et al. reported that protein methyltransferase-like protein (METTL)-3 can regulate adipogenic differentiation of BMSCs by regulating JAK1/STAT5/C/EBPβ signaling pathway [24]. Other studies have found that interleukin 6 receptor (IL6R) can promote adipogenic differentiation of human mesenchymal stem cells by activating P38 signal pathway [25]. Our team's previous studies found that aspirin can regulate the adipogenic differentiation of TSPCs through the PTEN/PI3K/AKT signal pathway [26], thus reducing the accumulation of adipocytes in the injured tendon. To clarify the internal mechanism of the difference of adipogenic differentiation ability of TSPCs between young and aging rats, we used RNA sequencing to explore the difference of gene expression between the two groups. According to the results of GO and KEGG, we selected PPARγ signaling pathway as the target pathway. First of all, we found PPARγ signaling pathway was inhibited, the gene expression of CD36, FABP3, OLR1, Perilipin, RXRA, Slc27a6 and the protein expression of PPARγ, Slc27a6, RXRA, angptl-4, perilipin were decreased. Then, in order to verify whether the inhibition of PPARγ signaling pathway led to the decrease of adipogenic differentiation ability in aging TSPCs, we added Rosiglitazone maleate, a specific agonist of PPARγ signaling pathway, to the adipogenic induction of aging TSPCs, and found that expression of PPARγ, perilipin and Slc27a6 molecules at the protein level were significantly higher than those of the control group. At the same time, the results of oil red O staining also showed that the adipogenic differentiation ability of aging TSPCs was significantly improved after adding Rosiglitazone maleate. These results indicated that adipogenic ability of aging TSPCs decreased due to the inhibition of PPARγ signaling pathway.

In our previous work, we found there were more adipocytes accumulated and adipogenic markers increased the expression in the tendon injury sites. However, in this research, we found that aging decreased the adipogenic differentiation of TSPCs, this could not explain the phenomenon that more TSPCs differentiated into adipocytes during tendon repair in the elderly, which leads to the accumulation of adipocytes. In this regard, we believe there are one or more factors that have stronger regulatory effects on TSPCs in the microenvironment of TSPCs. For example, a cytokine that can induce adipogenic differentiation of TSPCs expressed much more in aging rats than that in young ones. Therefore, further researches should be done to find reasons for the increased adipogenic differentiation of TSPCs in aging individuals. Niche is a specialized dynamic micro-environment which regulates stem cells’ functions and fate. Aging of cellular and acellular components of the niche can alter stem cell functions. Many studies have found that exposure of stem cells to young systemic environments can restore the ability of aging mouse stem cells to regenerate in different tissues [27, 28]. Transplantation of aging spermatogonial stem cells into the testes of young male mice can maintain its cell function [29]. These findings emphasize the important role of microenvironment in the decline of stem cell function, and propose to reverse tissue aging by targeting regulation of stem cell microenvironment. We believe that the decrease of adipogenic differentiation ability of TSPCs in aged individuals is mainly due to the decrease of “stemness” which is an internal factor of TSPCs during aging. However, as TSPCs reside in the tendon, they will be regulated by external factors. Therefore, cytokine microarray was used to screen the cytokines that were significantly different in the early stage of tendon repair between young and aging rats. The innovation of this study is to try to explore the potential cause of adipocyte aggregation of TSPCs in tendon tissue through comparative analysis of cytology and histology. In addition, we also clarify the specific mechanism of the decrease of adipogenic differentiation ability of TSPCs in the elderly.

## Conclusions

In summary, the present study demonstrated that aging inhibited adipogenic differentiation of TSPCs, and for the first time revealed the process was related to the downregulation of PPARγ signaling pathway. It is not likely that the adipocytes accumulation in aging tendon during repairing was due to the aging of TSPCs. And it is of great significance to find the cues in the TSPCs niche that influenced the differentiation abilities.

## Data Availability

All datasets used and/or analyzed during the current study are available from the corresponding author on reasonable request. The sequencing data is available on SRA database and the BioProject accession number is PRJNA673592.

## Abbreviation

TSPCs: Tendon stem/progenitor cells
MSC: Mesenchymal stem cell
qRT-PCR: Quantitative real-time polymerase chain reaction
PBS: Phosphate buffered saline
DMEM: Dulbecco’s modified Eagle’s medium
FBS: Fetal bovine serum
